# Do you wanna dance? Tales of trust and driving trust factors in robot medication counseling in the pharmacy context

**DOI:** 10.3389/frobt.2024.1332110

**Published:** 2024-08-07

**Authors:** Susanne Hägglund, Malin Andtfolk, Sara Rosenberg, Mattias Wingren, Sören Andersson, Linda Nyholm

**Affiliations:** ^1^ Faculty of Education and Welfare Studies, Åbo Akademi University, Vaasa, Finland; ^2^ Pharmaceutical Sciences Laboratory, Faculty of Science and Engineering, Åbo Akademi University, Turku, Finland; ^3^ Department of Caring and Ethics, Faculty of Health Sciences, University of Stavanger, Stavanger, Norway

**Keywords:** trust, medication counseling, human–robot interaction, socially assistive robots, pharmacy and medicine, medication safety

## Abstract

**Introduction:** The sustainable implementation of socially assistive robots in a pharmacy setting requires that customers trust the robot. Our aim was to explore young adults’ anticipations of and motives for trusting robot medication counseling in a high-stakes scenario.

**Methods:** Through a co-creation approach, we co-designed a prototype application for the Furhat platform together with young adults. In-lab testing of a pharmacy scenario, where the robot provides medication counseling related to emergency contraceptive pills, was conducted to deepen our understanding of some factors driving young adults’ initial trust establishment and anticipations of interacting with a robot in a high-stakes scenario. Qualitative data from interviews with six study participants were analyzed using inductive, reflexive thematic analysis and are presented through a narrative approach.

**Results:** We outline five tales of trust characterized by personas. A continuum of different anticipations for consulting a robot in medication counseling is presented, ranging from low to high expectations of use. Driving factors in the initial trust establishment process are position, autonomy, boundaries, shame, gaze, and alignment.

**Discussion:** The article adds to the understanding of the dimensions of the multifaceted trust concept, of driving trust factors, and of the subsequent anticipation to trust robots in a high-stakes pharmacy context.

## 1 Introduction

Advanced emerging technologies such as robots are becoming more common in the workplace, including healthcare (Hosseini et al., 2023). Socially assistive robotics (SAR) is the field of researching robots that assist users by way of social interaction (Trost et al., 2019). It has been suggested that the COVID-19 outbreak could herald an era of greater integration of robots in healthcare, as socially assistive robots could strengthen security by reducing human contact, thereby minimizing the spread of viruses (Yang et al., 2020; Zeng et al., 2020; Giansanti, 2021). Apart from the many benefits their introduction entails, ethical challenges such as privacy rights and the responsible use of technology arise. Concerns have been voiced that advanced robots may create responsibility gaps through a lack of clarity about who is responsible for the performance and result of a task, should a task that was previously performed by a human being be handed over to a robot, for example (Hosseini et al., 2023). Recently, calls have been made for caution and regulation of artificial intelligence (AI)-supported health technology (European Commission, 2023; Federspiel et al., 2023).

Simultaneously, the pharmaceutical field struggles with challenges. Prior research points to global shortages of care professionals, including pharmacists (Ikhile et al., 2018), and heavy workloads in pharmacies (Ljungberg Persson et al., 2023). This is reflected in downstream effects in medication processes, for example, medication errors occurring in 10%–20% of medication orders (Gates et al., 2019), poor quality of medication counseling (Alastalo et al., 2023), and poor handling of medication errors (World Health Organization, 2017; Ministry of Social Affairs and Health, 2022). Research is scarce on the topic of the pharmaceutical use of socially assistive robots, but a scoping review by Andtfolk and colleagues (in preparation, 2024) proposes that socially assistive robots may be considered suitable for use in medication processes. For example, socially assistive robot interventions such as medication advice have had positive results in prior research (Broadbent et al., 2018; Robinson et al., 2020), and Alahmari et al. (2022) state the need for robotic-assisted pharmacies to distribute drugs to eradicate or substantially reduce human error. However, pharmacies and medication counseling are strictly regulated fields. Regulation acts as a safeguard to prevent severe health risks due to medication errors, but these occur occasionally, nonetheless. The scoping review (Andtfolk et al., 2024a) indicates that using safe and trustworthy socially assistive robots in medication processes might have potential.

As socially assistive robots may be expected to perform more tasks in healthcare, understanding human trust in robots to carry out tasks is an essential research topic (Archibald and Barnard, 2018; Lyons and Nam, 2021; Schneider and Kummert, 2021). Trust is a critical element in human–robot interaction (HRI) scenarios, where humans rely on the robot to meet their goals in vulnerable and potentially high-risk contexts (Pinto et al., 2022; Saßmannshausen et al., 2022; Kumar et al., 2023). Moreover, as AI-based decision-making systems are being implemented, a requirement for trustworthy AI systems is that users accept and are willing to use and trust them (Kaur et al., 2022). Trust has a significant effect on the anticipation of using AI applications in the future and, thus, acceptance of AI technology (Hyesun et al., 2022). Moving beyond causality and effect, trust has been defined as being an anticipatory, continually emerging feeling that resides in the liminal space between the present and what we think will happen in the future (Pink, 2021). We approach this call for more research on trust in AI-enhanced technology such as robots by exploring Finnish community pharmacy (henceforth referred to as pharmacy) customers’ establishment of initial trust in robot medication counseling in the high-stakes scenario of purchasing emergency contraceptive pills (ECPs) through user testing of a prototype robot application, co-designed with end users and pharmacists.

Emergency contraception may prevent unplanned pregnancy after unprotected sexual intercourse if used correctly (Atkins et al., 2022). The mechanism of action is inhibition of ovulation to prevent fertilization (Rosato et al., 2016; Endler et al., 2022). In Finland, emergency contraception is often given in the form of pills and is available at pharmacies without age limit restrictions or a prescription (FSHS, 2023). However, some women have reported that buying ECPs has been uncomfortable. In-depth interviews with young women in London report experiences of pharmacy staff as judgmental, unsympathetic, and unsupportive (Turnbull et al., 2021). A survey (The Pharmaceutical Journal, 2018) found that some women would rather travel to another city to purchase ECPs to avoid meeting acquaintances at the pharmacy. In addition, the women said they waited until the pharmacy was empty of customers before asking about ECPs. This is consistent with the finding of Turnbull et al. (2021) that transactions in a pharmacy are not discrete and can jeopardize client confidentiality and privacy. Against this backdrop, this article serves as an exploration of customer experiences in a potentially vulnerable, uncertain, and awkward scenario where a socially assistive robot performs the tasks of medication counseling for ECPs.

The aim of our research was to explore some of the factors driving young adults’ choice decisions when establishing initial trust in a robot, and our work was guided by two research questions. The first one targeted choices that a young pharmacy customer might make regarding robot medication counseling for ECPs. To explore this first research question, we examined whether customers anticipated trusting medication counseling to the robot (RQ1). The second research question focused on motives driving the choice to trust or not trust the robot. We aimed to explore how young adults perceived the robot, indicating factors that either drive or impede trust in robot medication counseling (RQ2). The aim of the article is to contribute new knowledge to the field of human initial trust establishment in the context of trust in robot medication counseling in pharmacies.

## 2 Theoretical background

### 2.1 Trust in a human–robot interaction context

Currently, there is no widely accepted definition of trust and its conceptualization, nor is there a methodological paradigm for its evaluation (Law and Scheutz, 2021; Saßmannshausen et al., 2022). In previous HRI studies, the trust process has been related to the attitude or belief of the trustor in the sense that the trustee—the robot—will help achieve the goals of a trustor—for example, a pharmacy customer—in an uncertain and vulnerable situation (Lewis et al., 2018; Rossi et al., 2018). Trust has also been explored as the subsequent behavior of this belief or the extent to which one chooses to rely upon recommendations or actions of the agent (Lewis et al., 2018; Schneider and Kummert, 2021). Falcone et al. (2023), on the other hand, argue that trust can simultaneously be a mental attitude toward an agent, a decision to trust it—rendering the trustor vulnerable—and a behavior, an intentional act to trust the robot. Hancock and colleagues have defined trust as “an individual’s calculated exposure to the risk of harm from the actions of an influential other (2023),” whereas Lee and See (2004) suggest the following definition: “the attitude that an agent will help achieve an individual’s goals in a situation characterized by uncertainty and vulnerability.” This study adheres to the latter definition, as the scenario includes ECP purchases that are most likely characterized by uncertainty and vulnerability. We have operationalized initial trust establishment as attitudes toward the robot and its task in this vulnerable situation. The attitudes and reasoning regarding trusting the robot and the medication counseling are explored through individual semi-structured interviews post human–robot interaction.

Several factors may influence a human trustor’s belief in the robot trustee’s benevolence and capability to act in a risk-mitigating manner (Hancock et al., 2023). Recently, trust in HRI was conceptualized as a quadratic model where humans, robots, the environment, and interaction/communication function as antecedents of trust (Saßmannshausen et al., 2022). Trust is a multifaceted concept encompassing many dimensions (Pinto et al., 2022), and thus, its assessment in HRI is challenging, partly because trust is multidimensional and context and culture dependent. Trust is also impermanent and fluid, as it is continuously recalibrated over time as prior experiences of HRI are complemented with new knowledge and experiences (Pinto et al., 2022; Saßmannshausen et al., 2022).

### 2.2 Trust in healthcare

Trust is a care value manifested by actions and tasks in care practice (van Wynsberghe, 2017). Its importance in healthcare is well-documented and remains a mediating factor in the effectiveness of healthcare provision (Douglass and Calnan, 2016). General social trust levels are very high in Finland, being part of “Nordic exceptionalism” (Lundåsen, 2022), and trust levels in Finnish healthcare are relatively high (Keskimäki et al., 2019). Healthcare in Finland performs well in terms of trust as it ranked second in trustworthiness ratings in the EU in the spring of 2021 (Eurofound, 2020).

Only 18% of Finns aged 25–34 stated that they trust AI-powered services in general, and only 35% of that age cohort feel they have been informed about the safe use of AI-powered services, with 59% wishing to be better informed on the topic (Finnish Digital and Population Data Services Agency, 2023). The rationale of our study is that despite, and precisely because of, the complexity and volatility of trust dimensions at play, it is worthwhile exploring driving factors motivating choices when discussing the potential introduction of socially assistive robots in vulnerable scenarios such as purchasing emergency contraceptives. Understanding drivers and manifestations of trust in HRI may determine the implementation of sustainable and ethical robot applications in social systems such as pharmacies.

### 2.3 Mediation

Several theories posit that there are complex, casual, and multidirectional relationships at play within a social system (Norman, 2023). Some theories that have been applied in a care setting are theories of action with an explanatory focus on how new technologies, ways of acting, and ways of working become implemented in everyday practice (such as normalization process theory; see May et al., 2022), whereas others focus on individual users’ ascribed attributes and characteristics of a technology (diffusion of innovations theory, see Rogers, 1995). Other theories take a more descriptive and ethnographic approach to human agency. For example, the sociotechnical systems (STS) framework argues that technology is shaped by the social and cultural context in which it is implemented. Hence, it emphasizes a need to examine this interplay between the social and the technical and how technological developments are shaped by broader social structures and, in turn, shape themselves. From a sociotechnical perspective, successful implementation of technology within an organization is dependent upon the joint optimization of social components, like peoples’ needs, attitudes, knowledge, and skills, and the (emerging) technology in question (Winter et al., 2014; Norman, 2023).

Another theory that focuses on the interplay and the mutual shaping of the technical and the social is the technological mediation theory (TM). Building on post-phenomenological studies, the theory emphasizes an empirical focus and takes the design and development of technology as its starting point for analyzing relationships between humans and technical objects. Technologies are viewed not as instrumental objects but as mediators of human experiences and practices, thus actively shaping relationships between humans and the world (Verbeek, 2016; Nyholm, 2023).

TM does not recognize sharp distinctions between humans and the technologies that they use and favors a blurring of the boundaries between them, much in line with actor-network theory (ANT) (Verbeek, 2013). In contrast to ANT, however, TM addresses the hermeneutic dimension of mediation as it explores ways in which technological artifacts actively participate in shaping humans’ interpretations of the world while humans interact with them. Technologies participate in changing humans’ perceptions, experiences, values, and actions and thus also mediate morality (Verbeek, 2016; Kudina and Verbeek, 2018). This theory’s advocates stress that the dynamics of the interaction between human values and technology is best explored empirically and “in the making,” that is, grounded in micro-level practices of design and actual use, rather than being speculated about and assessed through ethical frameworks (Verbeek, 2016; Kudina and Verbeek, 2018).

Thus, a technological mediation approach proposes a way to understand how people anticipate their engagement with and normative implications of experimental technologies not yet implemented on a large scale as a part of a learning process (Kudina and Verbeek, 2018). Accordingly, TM has the ambition (Verbeek, 2015; Verbeek, 2013) to inform interaction designers in their work adhering to design thinking or value-sensitive design (see, for instance, integrative social robotics (Seibt et al., 2020) and care-centered, value-sensitive design (van Wynsberghe, 2017).

There are several pitfalls in HRI design to be aware of. Robots are often designed as either a thing or a being. However, they are often perceived by humans as both, and as neither of these but as an Other with psychological and social superpowers that offers the possibility to design a new hybrid, ambiguous entity (Dörrenbächer et al., 2023a) that mediates with assistive functions in social settings (Kaerlein, 2023). Another pitfall is, according to Dörrenbächer et al. (2023a), the urge to imitate human–human interaction in the design of robots. Such an approach fails to acknowledge that robots act as our counterparts instead of tools that extend human abilities. The authors argue that robots and humans constitute each other through diverse otherware relationships that depend on the mode of the interactivity involved (Dörrenbächer, 2023b, 31). Participatory design methods may assist robot designers in understanding how people anticipate coexisting with robots and in shaping the coexistence of humans and robots (Dörrenbächer, 2023b).

Knowledge about human social interactions with robots has been described as “still dimensionally incomplete” (Seibt et al., 2020), as research in the field of SAR has not sufficiently explored ethical, cultural, or existential aspects of peoples’ experiences of interacting with a socially assistive robot. Seibt et al. (2020) call for using items in the humanities research toolbox, such as phenomenological analysis and narrative analysis, to address the need for more knowledge on human social interactions with robots.

In our analysis, we draw upon technological mediation theory and a value-sensitive design approach to further knowledge of the interrelationship between customers and socially assistive robots in a pharmacy context. We present observations of anticipatory concepts of robot futures through five personas that may inform and guide design and research in the field. We chose a narrative approach to address the call for multidimensional descriptions of HRI. Seibt et al. (2020) suggest narrative analysis methods to counteract what they call the description problem in social robotics, where humans’ social relationships with robots are explored too narrowly. De Pagter (2022) recommends a more explicit inclusion of a narrative focus to come to grips with how trust building between humans and social robots can be further developed. Given the small sample size of the study, a narrative approach may capture the nuances of the initial trust establishment phenomenon and ultimately contribute to the understanding of what the trust-building process may look like. We hope that our analysis of the six shared informant experiences as five personas may contribute to this. As noted above, trust has been identified as one important driver of successful interactions between humans and robots, but more in-depth studies are called for on the topic of antecedents of trust and human interaction with robots (Saßmannshausen et al., 2022). Therefore, as a step in a humanity-centered design process (Norman, 2023), we aim to contribute new knowledge on initial trust establishment in robot medication counseling in pharmacies.

## 3 Methods and materials

The study is part of a multi-disciplinary PharmAInteraction project exploring whether socially assistive robots at pharmacies may strengthen patient and medication safety (cf. Andtfolk et al., 2022; Andtfolk et al., 2024b). The article draws on data from the first stages of a design process where we iteratively co-design, develop, and test a prototype robot application with potential end users and pharmacists.

### 3.1 Study stimuli

In Finland, ECPs are over-the-counter medication, but they require medication counseling concerning side effects, function, use, and individual needs. In our study scenario, the socially assistive robot Furhat was assigned the role of a standalone Sweden-Swedish-speaking ECP counselor with no pharmacist in the immediate loop (cf. Hagglund et al., 2023 for a video of the interaction flow of the application).

As medication counseling is strictly regulated, we designed a controlled system based on rules instead of an adaptive decision system. The application presents different options based on speech input from the customer concerning the following variables: hours since unprotected sexual intercourse, allergies, underlying diseases, and regular medication. The manuscript for this interaction flow was created together with pharmacists (Using role play and Hierarchical Task Analysis, 2023). Based on the user input to these queries, Furhat presents available options for medication and informs the customer on the function, use, and potential side effects of these options. Lastly, the customer is given the opportunity to choose between several available options. The robot provides the study participant with information about the range of available substitutable alternatives and pricing; communication of this information is mandatory according to national legislation (Finlex, 1987).

### 3.2 Participants and data collection

Purposeful sampling (Palinkas et al., 2015) was the chosen technique for recruiting participants who met the following criteria: being Finland-Swedish speakers, within the age range of 20–40 years, identifying as female, and having some experience using technology. Prior experience of or attitudes toward ECPs was not a criterion for inclusion and was not explored. Six study participants (all identifying as female) agreed to participate in the role-playing study evaluating the prototype robot application created as study stimuli. The research was carried out in a laboratory setting in Ostrobothnia, Finland, with participants from the area, having Finland-Swedish as their primary language, aged between 29 and 37 (mean age 30.5) and being somewhat experienced with technology (mean 3.83 on a scale of 1–5, where 1 corresponds to the least and 5 the most), though none had previously met Furhat. Study design rather than achieved data saturation governed the extent of the data collection phase. All six study participants had previously, approximately 2 months earlier, role-played the same scenario in the same laboratory setting with a pharmacist serving them as customers. Thus, the study participants reported here also participated in the previous scenario with a pharmacist. Therefore, playing the role of a customer buying ECPs was familiar to them, as was the multi-disciplinary research team and laboratory setting. This article, however, reports the experiences of the condition where Furhat provided medication counseling instead of a pharmacist.

**FIGURE 1:**
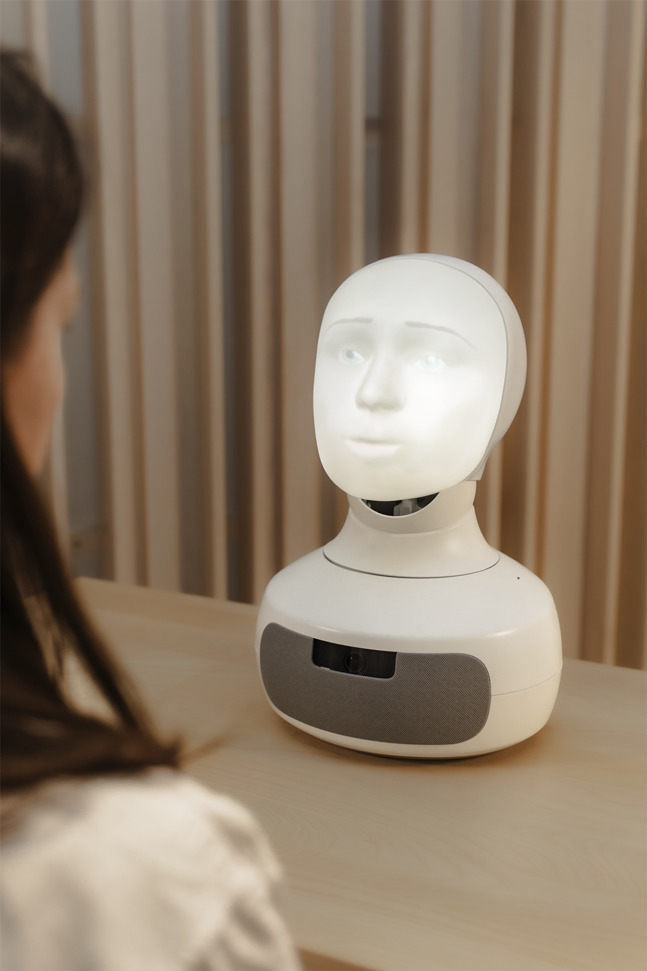
Study participants individually role-played a scenario with Furhat in a simulated pharmacy. Staged photograph by Marie Lillhannus.

Study participants met Furhat individually in a simulated in-lab pharmacy (see Figure 1). First, we went through the consent to participate forms and privacy notices. Then, we assigned the participant a role as a customer (see Supplementary Appendix S1 for task and scenario description). Next, the participant entered a room for robot medication counseling. A member of the research team was present in a rear section of the room in the event of technical challenges. The human–robot interaction was video recorded for analysis and will be reported elsewhere, as this article focuses on qualitative interview data. After interacting with Furhat, the participant exited the simulated pharmacy. Lastly, qualitative data were collected through six individual semi-structured post-test interviews, ranging from 24 min to 46 min (mean length 32:27 min), and recorded for analysis (see Supplementary Appendix S2 for interview guide). As the study participants had now role-played the scenario with both Furhat and a pharmacist, the interview guide focuses on the experiences of the two occasions.

### 3.3 Analysis

Data were analyzed using a process of inductive, reflexive thematic analysis (Braun and Clarke, 2022), with a focus on broad thematic patterning across all data. Analysis was conducted from a constructivist perspective. Reflexive thematic analysis is a method that integrates well into co-creation studies due to its flexibility and accessibility (Braun and Clarke, 2022, p 261).

Our reflexive thematic analytic approach began with familiarizing ourselves with the data by reading through the interviews multiple times. Three of the authors (SH, MA, LN) worked together to increase the validity and reliability of the analysis. A reflexive and iterative strategy was used during the coding process, where our preunderstanding, reflections, and interpretations were discussed within the group. This approach enabled a deeper understanding of the participants’ experiences and the underlying thematic structures. The coding was adjusted in multiple iterations, resulting in over one hundred codes of potential analytic interest. Subsequently, codes were organized into broad themes, which related to what a robot is, the interaction with the robot, the experience of non-judgment, and the importance of autonomy.

After reviewing the codes and the whole dataset, we became most interested in the latent idea underpinning articulations related to trust, as we noted that all patterns somehow related to varying degrees of trust. We identified factors in the codes that are driving motives when establishing and calibrating one’s trust in the robot and its use case. These trust factors or motives act as subthemes and address the second research question. The anticipation to trust the robot or not became a set of five distinct themes, ranging from low to high levels of trust, that address the first research question. Each theme—articulated as a song title—captures a different expression about trust in relation to robot medication counseling, and we treat these metaphors (Lakoff and Johnson, 1980) as personas (Lewrick et al., 2018). We chose song titles for the themes as the process of establishing initial trust in a novel robot appears to resemble a dance. We interpret the dynamic interaction between the robot and study participants and the subsequent interview stories regarding anticipations to trust the robot with providing medication counseling as motion and continuous calibration. The organized codes revolved around finding space, a shared rhythm, appropriate forms, and quality performance, among other things, which steered our thoughts to elements of dancing. The themes are illustrated through personas, which is a common concept in interaction design (Lewrick et al., 2018).

The findings are presented with illustrative quotes from the participants that capture the driving motives for intentions to interact with the robot.

### 3.4 Ethical considerations

The procedures used in this study adhere to the tenets of the World Medical Association’s Declaration of Helsinki (2022). Ethical research practices (TENK, 2023) were applied, participants provided their written informed consent to participate in this study, and data were pseudonymized. Participation was voluntary, and all study participants were informed of the possibility of withdrawing from the study without providing any further explanation. According to the Finnish National Board on Research Integrity TENK (2019), the study design does not warrant an ethical review statement from a human sciences ethics committee. The study participants were not real pharmacy customers in actual need of emergency contraception in the role-playing study, but they represented their sociodemographic group of young women aged roughly 18–40. They were not at risk of physiological health injuries as they role-played a scenario in a laboratory without any medication present.

Furthermore, our work relates to ethics in a methodological way. The practice of designing with and by, instead of *for*, users is well-established in today’s design field (Norman, 2023) and increasingly so in HRI technology development (Ostrowski et al., 2020). The method of co-designing is an ethical orientation, with its focus on inclusion and equality (van der Velden and Mörtberg, 2014; Carros et al., 2023). Technological mediation theory defines technology design as experimental ethics and argues that ethics benefits from exploring actual practices of design, use, and implementation of technology. Instead of “merely” assessing technology from the outside, Verbeek (2013) argues that ethics should come up with new ways of doing ethics of technology empirically and from “within.” Applying design thinking methods has also been suggested to provide insights into whether and how technology and the social practices it affords lead to ethically desirable or virtuous practices (Misselhorn et al., 2023).

## 4 Findings—tales of trust and driving factors

**FIGURE 2:**
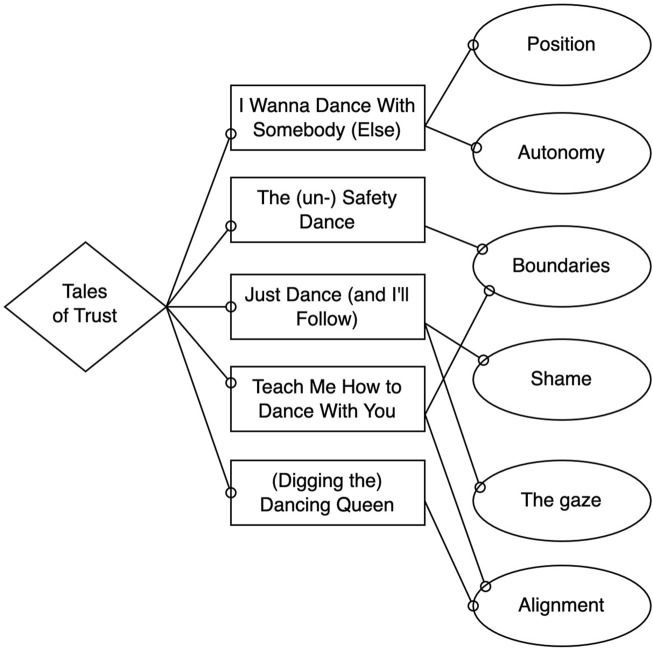
From left to right: Main theme, themes, and subthemes. The links between subthemes and themes indicate the main driver(s) in the persona’s trust process.

In the two research questions, we explored whether the study participants anticipated trusting medication counseling to the robot and how the young adults perceived the robot, indicating factors that either drive or impede trust in robot counseling. The main theme of the analysis is Tales of Trust, illustrating the varying outcomes of the dynamic HRI (see Figure 2).

The findings in this study show that the study participants’ anticipations of using socially assistive robots as a source of information within the pharmacy context vary considerably. Some participants expressed low acceptance of the use of robots as a source of information as they mistrusted the robot, while others saw great potential. Trust, in turn, shaped behavior in the robot simulation. Song titles with meaningful content are used as metaphors to illustrate our tales of trust and strengthen the five themes of the analysis. Each theme exemplifies the acceptance of the participants, progressing from the more skeptical to the predominantly positive perceptions. The six study participants are clustered into five personas or fictional yet realistic characterizations. The persona Just Dance (and I’ll Follow) merges two similar tales of trust and thus represents two study participants.

The subthemes (see Figure 2) illustrate important factors driving trust in this particular robot behavior and use case, as raised by the participants. The figure contains links between some subthemes and themes, illustrating what we found to be the salient main driver(s) of the personas’ narratives. For example, the persona The (un-)Safety Dance did discuss the gaze behavior of the robot as an example of anthropomorphic robot cues. However, we interpret her total experience as being more about the boundaries of humans and robots and whether the robot is a tool or an agent than about being observed by a robot’s gaze. Thus, although several subthemes may have been expressed by a persona, we choose to highlight the one or two we interpret as most predominant in the analysis.

### 4.1 I wanna dance with somebody (else)

Neither the robot itself nor the use of it as a source of information is of particular interest for this persona. She values being independent and, therefore, seeks necessary information about medication on her own. As she does not trust the information the robot gives her, she would like to verify it with a pharmacist: *I would have gone to a pharmacist and asked, “Did I get this right?”* She appreciates the freedom to choose and wants to decide for herself with whom she wants to interact. She does not see the robot as an agent but rather as a tool that should be controlled: *I can imagine that this could be a bit like self-checkout at the grocery store, where you can choose to take the fast route: go to the robot, get the information, and go home.* She is also very skeptical of the environment the robot is in, as the location affects her trust in the robot. In her opinion, only healthy people could benefit from robots. Furthermore, she does not trust that the robot recognizes her intentions, as people can lie to it: *Perhaps one might give the robot an answer that may not be entirely truthful because one just wants to move on to the next question.* If she still must interact with a robot to get the medication, she wants to choose the robot’s attributes, such as appearance.

This persona dismisses interacting with the robot and hurries away from the robot dance floor toward another disco.

### 4.2 The (un-)safety dance

This persona is interested in the robot but is uncertain about its competence. According to her, the robot did not respond adequately to her questions, which diminished her trust in the robot’s competence. She wishes the robot were more responsive and capable of handling emotionally charged situations. According to her, the robot looked pleasant but did not behave like humans do. The non-human facial expressions and gestures further weakened her trust: *I think it wandered with its gaze, which made me feel a bit, you know, not on guard but rather uncertain.* Therefore, she is not convinced that she can rely on the medication information provided by the robot. She suggests that this could perhaps be alleviated through additional information, such as written or displayed content. If she is going to speak with the robot, she calls for clear instructions on how to communicate with it: *How can I convey this information in a way that the robot picks up what I mean; how can I be clearer?*

This woman prefers to be served by a human rather than a robot. Trusting a robot requires more of her than trusting a human being does. She suggests that the robot would function better by working as a team with a pharmacist. She would feel more secure if a pharmacist were present in the background, ready to intervene if something goes wrong: *It would also be good if there was a button to call for staff to come and assist.* The woman needs a human to verify that the medication is suitable and safe, specifically for her.

This persona cautiously dances one dance with the robot and then chooses to stand against the wall, attempting to gauge the reactions of others on the dance floor. She wonders if the robot is a tool or an agent.

### 4.3 Just dance (and I’ll follow)

This persona emphasizes that the robot has certain advantages over a human, the main one being that one does not need to feel ashamed in front of a robot. The robot does not judge, even though the matter is sensitive in nature. *You might not want to see a pharmacist because you know you shouldn't be in that situation in the first place.* Especially in a small community, she prefers avoiding being seen. For her, interacting with a robot grants invisibility. This means she does not have to expose herself to a human, and the robot protects her privacy. *With the robot, I felt more at ease because I can answer anything.* However, she wishes the robot would communicate more quietly so that the people nearby cannot overhear the nature of her errand. She suggests that perhaps it could be placed in a secluded area to ensure her privacy.

After interacting with the robot, she spends a long time contemplating its gaze. She perceives the robot’s gaze as neutral and non-judgmental, which increases her trust. Its gaze does not carry the same weight as that of a human. *While the robot also maintains eye contact, it feels different than when a human does so.* The woman suggests that she can let go of her façade and relax when being served by a robot. It feels good not to have to wonder what the robot is thinking because, well, it does not think. The rules of social interaction are thus set aside. Additionally, the robot is always equally agreeable to all customers, unlike humans. This woman emphasizes that there is no risk of being offended by a robot.

This persona enjoys dancing with the robot as it allows her to avoid dancing with a human. By doing so, she avoids the risk of being judged.

### 4.4 Teach me how to dance with you

It is exciting to interact with a robot, according to the persona in this tale, and she is eager to try it out. It feels a bit challenging because it is her first time speaking with a robot. *And it was also nerve-wracking, but I believe that over time, I might become more comfortable with it*. When she notices that the robot does not recognize what she wants to say, she becomes uncertain and feels insecure, rejected, and frustrated. She questions her own way of interacting due to these communication failures. She wants to learn how to interact with a robot in the best way possible, and she wonders how she can adapt to the robot*. I noticed that I improved my language with the robot, but maybe, yes, I wanted it to understand me, so I started to articulate a little more and speak using complete words so that it would understand.* She wishes for clear instructions on how to communicate with a robot.

It is important that the robot does not interrupt her; she wants to convey her message without haste. The robot should be as responsive as humans, as that would enhance her perception that the robot’s responses are tailored specifically to her. The choppiness in the dialog flow negatively affects her sense of trust in the robot. The robot should not speak too quickly, and it is beneficial to repeat information about the medication when necessary *… because I didn’t quite understand all the information the robot provided, so I would have liked it to repeat its message.* She also wishes for a clear confirmation, affirming that the robot has processed her message. She calls for such a confirmation in the form of a repetition of her message by the robot.

The persona in this tale wants to learn how to dance with a robot. She tries to adapt to the robot’s steps but becomes disappointed when the dance does not turn out the way she envisioned. She wonders if the robot is a friend or foe.

### 4.5 (Digging the) dancing queen

This persona is enthusiastic after interacting with the robot, and she sees great potential for robots offering medication counseling in the future. She has many questions about the robot’s performance. She trusts the information she receives from the robot, and she feels that the information provided is consistent: *It feels very reassuring that I’m getting all the information I need, that I can trust the information I receive.* She expects to be heard by the robot and to be taken seriously. However, she wishes that the robot were more flexible, would ask additional follow-up questions, provide spontaneous information, and wait for her replies instead of moving along without considering her input. In this story, the woman seeks a dialog on ontological questions and wonders how robots and humans actually differ.

The woman appreciates the robot’s human-like appearance, and she is surprised by how important the robot’s appearance is to her. She appreciates the opportunity to interpret the robot’s gender role: …*and very androgynous, not directly a woman or a man, but you can interpret it as you need.* The woman longs for an even more human-like interaction with the robot: *It could be more nuanced in the language, not always responding with “oh, so you said … ” but varying, for example, with an “mm,” just like we do “mmm.”* The voice could also be more human-like, according to her. She also calls for robot confirmation and acknowledgment of what she has said, much like when drive-thru clerks repeat her order, for validation and to avoid misinterpretations. She feels it is her fault if the robot does not acknowledge what she says and alters her way of speaking to it.

In this last tale, the persona thoroughly enjoys being on the dance floor with the robot. She does not miss dancing with humans, and she hopes that the robot can become an even better dance partner.

## 5 Discussion

The findings outline study participants’ drivers of trust and subsequent choices to trust medication counseling to a robot. Five themes are summarized in the tales of choices, ranging from low to high levels of trust and acceptance, and underlying motives are presented as six subthemes. In the following, we will discuss the factors driving young adults’ choice decisions when calibrating trust in a robot and the interplay between the motives and succeeding choices to trust the robot with medication counseling.

### 5.1 Position

The persona I Wanna Dance With Somebody (Else) is quite reluctant to interact with the robot. The tale highlights the importance of position, both in terms of physical and/or structural environment and medical circumstances. Regarding the former, protecting the *integrity and privacy* of the customers interacting with the robot is required. Careful planning of the physical placement, the establishment of interaction safe zones, and multipurpose labeling of the robot help to minimize the risk of other customers learning the nature and details of sensitive errands. The concerns also extend to the digital realm. The uncertainty of personal data governance breeds insecurity, as voiced by the third persona. This concern is in line with recent study results reported by the Finnish Digital and Population Data Services Agency (2023), showing that only 21% of young Finns aged 25–34 trust safe data privacy management in AI-empowered services. Earlier studies have showcased the importance of data and integrity protection for emergency contraceptive customers (Turnbull et al., 2021), as these data suggest as well. Protection of privacy and (data) integrity is one of the make-or-break drivers of trust expressed in our study, echoing the findings of frameworks (Kaur et al., 2022) and earlier studies, where integrity is a suggested component of moral trust (Malle and Ullman, 2021) and organizational trust (Pearson et al., 2016).

As a complement to the physical environment, the study participants discuss their position in terms of medical circumstances. The analysis shows how the *complexity* of the situation affects the willingness to consult a robot for medical counseling. Examples include whether the medication is new to the customer, whether the customer has complex health issues they wish to discuss with a care professional, the level of confidence that the robot has correctly received and processed their message, and the perceived accuracy of the robot’s reply. Data include opinions that only healthy people benefit from HRI and a need to verify the robot’s advice with a pharmacist, who is more trusted than the robot, to be reassured that the medication is suitable and safe. If the medication is familiar, and there is no need for consultation or answers to questions, then the robot has the potential to serve as a trustworthy, smooth, and quick self-service technology. As complexity increases, study participants’ trust in the competence of a robot providing medication counseling—a high-stakes use case—is challenged. Thus, the participants perceived risks, fueling mistrust in the robot and greater confidence in pharmacists. Trust is partly explained by risk perception (Malle and Ullman, 2021; Pinto et al., 2022), and risk assessment lies at the very heart of the concept of trust.

### 5.2 Autonomy

The five tales highlight two aspects of autonomy that the study participants value in the interaction: *empowerment and independence, as well as freedom of choice* regarding the form of service. Both the least and the most interested persona in the HRI in this scenario value being able to search for information about the medication themselves. This implies an empowered role as a customer, shifting the task from the pharmacist to the customer. Robots are not necessarily trusted with retrieving this information, as the tales illustrate. One participant shared how disempowered she felt when the robot advanced in the dialog without taking her input about allergies into account. This challenges her ability to control and play an active part in health-related issues, which may result in severe consequences.

Another side to the empowerment coin is being independent as a customer. The ability to treat the robot as a quick self-checkout that limits time spent at the pharmacy could be valuable, should the situation allow for it, for example, when the medication is already familiar, and there are no health changes to consider. Lastly, the value of choosing the agent or counterpart to interact with each time is highlighted in the tales. Depending on the situation, a robot may be trusted to inform, whereas at other times, a human is preferred, and the ability to choose is valued.

These findings point to interesting shifts in perceptions of agency in the medication counseling process and pharmacy setting in general. The robot in the study scenario changes how medication processes could be carried out at pharmacies, dividing the tasks and responsibilities between a pharmacist and a robot. This shift has implications for customers’ behavior in and experiences of the pharmacy. The robot thus carries the potential to shape the practices of interacting with pharmacists and buying over-the-counter products. As the five main themes described above illustrate, a robot is likely to shape the anticipated actions differently, as some participants were more interested than others in consulting the robot, should it be available for ECP counseling. The participants want to make informed decisions and see themselves as active agents that co-create the experience and service instead of a customer entering the pharmacy and receiving a product from a care professional. The call for the ability to choose between human and robot assistance when entering the pharmacy informs the design of the service and localities and is likely to shape social norms impacting agents at the pharmacy as well. Batalden et al. (2016) note that, over the years, healthcare has transformed into a service cocreated by healthcare professionals together with people seeking help to restore or maintain health for themselves and their families. Trusting a robot to play a role in the co-productive partnership at a pharmacy should address the value of an empowered role of the customer cocreating the experience and service while carefully mitigating the risks of responsibility gaps following a lack of clarity about who is responsible for the performance and result of a task (Nyholm, 2023).

### 5.3 Boundaries

The tales reflect a continuum of experiences where, at one end, the robot is perceived as a tool to control, allowing for little acceptance of a robot in such a high-risk use case. In these tales, the professional team acting out the role of pharmacists (Goffman, 1959) does not include a robot for anything other than merely instrumental tasks, at best. At the other end, the robot is perceived as an agent on par with pharmacists, with clear benefits over humans; a robot never has a bad day or offends or shames the customer. In between, The (un-)Safety Dance persona expresses a trust default in humans, as she says that engaging in a risk evaluation of a robot requires much effort on behalf of the trustor. Calls are made for more emotional intelligence and responsivity in the robot, allowing for more human-like behavior. Here, a more “human interplay” and an ability to handle distress in the client serve as drivers of trust. However, human participation through *human-in-and-over-the-loop* (Kaur et al., 2022; Shneiderman, 2022) is considered a benefit by all but one persona. The presence of pharmacists is a requirement, albeit with potentially different tasks in the workflow and with limited proximity to the customer. This is in line with the findings of Alahmari et al. (2022), which stated that pharmacy robots are unable to entirely replace human duties.

Taken together, those study participants who are least accepting of robots tend to consider the robot as a tool, and the ones the most accepting consider it to a greater extent as an agent. All personas engage in an assessment of who the interlocutors in the dialog are, ontologically and socially, in relation to each other and their boundaries. Notably, the persona Teach Me How to Dance With You struggles with boundaries and how she should adapt for the dialog to run smoothly. Therefore, our analysis suggests that knowing who and what the robot is, ontologically, as well as in relation to the person interacting with it, is involved in driving trust. Knowing what the robot can do for the human without risking harm is another cofactor that drives trust. Consequently, lacking that piece of information fosters feelings of uncertainty and risk aversion.

Emerging drivers of trust also include explainability and accountability. The former concerns clarity regarding what the robot is, how it works, and *transparency of its competence.* The latter concerns a shared responsibility between the robot and the customer to avoid harm. The participants say that a crucial dimension of trust for them is accurate information or knowledge in this high-stakes scenario so that they can make an informed decision. Therefore, the robot must be competent to appear trustworthy, and currently, few feel it does so fully. In addition to the requirement for greater capability in medication counseling and answering questions, some participants suggest mitigation mechanisms to avoid harm and decrease uncertainty. Seeing the information on another non-robot interface is suggested, perhaps on a touchscreen where clients can see the packet of the suitable ECP or the list of its side effects. Our data are thus not in line with findings (e.g., Schneider and Kummert, 2021) that robots are trusted more and considered to be more competent the more autonomous they are. The calls for shared responsibility and increased user control to make informed decisions are interesting and may reflect the high-risk scenario where misunderstandings could lead to dangerous risks.

Our analysis thus identifies both relation-based and performance-based trust factors, and this is in line with earlier research on what constitutes trust. Law and Scheutz (2021) identify performance-based as well as relation-based dimensions of trust. The reliability, competence, and capability of the robot to successfully carry out tasks lie at the heart of performance-based trust. A vulnerable human being trusting a robot to be a social agent constitutes the moral/relational dimension. Our analysis showcases the suggested dimensions of performance, as well as relationship, in the initial establishment and calibration of trust and extends the notion to include an ontological dimension as well. A simple one-dimensional continuum between humanness and robot-ness does not mirror how humans reflect on robots and their differences from human beings (Ullrich et al., 2020). In that conceptualization, clashes may occur between new ontological categories, such as non-living and animate technologies much like robots, and the human moral-cognitive system (Laakasuo et al., 2021). The negotiation of humanness and robot-ness expressed by the study participants is in line with the observation that people find robots to be their counterparts, an Other or Otherware, rather than extensions of themselves (Dörrenbächer et al., 2023a).

### 5.4 Shame

The data show the value of *not having to feel ashamed or judged* while purchasing ECPs. Interacting with a robot for medication counseling might enable young customers to maintain a sense of self-respect and not feel ashamed about their situation, as illustrated by the persona Just Dance (and I’ll Follow). According to self-determination theory, human behaviors are both intrinsically and extrinsically motivated. Extrinsic motivation drives behaviors carried out for reasons other than inherent satisfaction, but these are still partially internalized through an avoidance of anxiety, shame, or guilt. The avoidance of shame functions as an internal reward of self-esteem (Ryan and Deci, 2020). Moreover, humans run the risk of shame and stigma (Goffman, 1963) in everyday interactions as we present ourselves to others on a social stage, influenced by social norms and values (Goffman, 1959). The finding of this study suggests the possibility of the robot acting as a mediator, offering the customer a chance to maintain a sense of self-esteem and to avoid having to search for approval from oneself or feel ashamed about their situation. The robot could be an example of a mediating technology (Verbeek, 2016) that may change the experience of an interaction to a less shameful or embarrassing one. Using Goffman’s dramaturgical metaphor of the theater (1959), the customer may find it beneficial to present themselves to a robot instead of a pharmacist on the pharmacy stage to maintain a sense of self-esteem. The robot in this particular scenario might help the young customer shape the experience and situation in a more positive way, diminishing the negative emotion of shame. Previous research has found that humanoid robots can elicit feelings of shame in human interlocutors (Menne, 2017; Schneeberger et al., 2019). In contrast, Pitardi et al. (2022) argue that service robots reduce potential customer shame to a higher degree than service employees. For some customers, robots could be the preferred means of service delivery in potentially embarrassing service encounters, including healthcare contexts. As the body of literature on shame in HRI is still small and points to complex interplays (Menne, 2017), our finding on the robot’s potential to alleviate feelings of shame and judgment contributes to the discussion.

### 5.5 The gaze

Interestingly, gaze is a concept that was found to both diminish and increase trust in our study. However, gaze is a broad concept. Two of its dimensions were discussed by the study participants, yielding opposing findings. On the one hand, gaze is a crucial mechanism in HRI that affects interaction through the process of joint attention, where the human and the robot share attention to reach a goal, and gaze aversion is a social cue in the interaction (Koller et al., 2022; Irfan et al., 2023). This dimension of gaze is expressed in this dataset. On the other hand, *gaze embodies values and opinions* between an observer and the observed. The personas refer to pharmacists’ judging of young customers through non-approving gazes, mirrored in the robotic gaze that is not perceived to contain such a moral judgment of the customer.

When the robot gaze behavior was perceived by study participants as being non-aligned with the expected and interpreted anthropomorphically, the sense of trust diminished, and feelings of insecurity as to whether the medication counseling was accurate and reliable arose. Earlier studies have found that robot gaze behavior matters in HRI (Skantze, 2021) and is a determinant of human trust (Babel et al., 2021). A recent study carried out on Furhat found that it is important that the gaze behavior is aligned with and grounded in the ongoing dialog and other parallel cues for HRI to be successful (Irfan et al., 2023). Our study extends this finding as it links robot gaze behavior to trust and echoes the importance of consistent robot gaze behavior for a successful interaction.

Concerning the content or the message of the robot gaze, we share findings that the study participants perceive it as neutral, non-judgmental, and inoffensive. Talking to the robot instead of a pharmacist allows the customer *to avoid being seen* and is a means of protecting one’s privacy and integrity. As the persona Just Dance (and I’ll Follow) highlights, HRI offers a chance to avoid being judged by the staff and other customers in the pharmacy. Many aspects of relief are expressed as strengths of HRI, such as the absence of prejudice by staff and exposure to the approval of staff and other customers. Other examples include the absence of human-to-human contact, which removes the need to maintain a social façade and to expose oneself by sharing private and sensitive matters with strangers and potential acquaintances. The robot is entrusted with private health issues, and non-judgmental treatment of the customer is expected. The quotes suggest that the robotic gaze does not convey as much meaning as the human gaze does, which is experienced as a positive thing in this particular case. Similar to the previous discussion on shame, the robot could help a young customer shape the experience to become more comfortable. This could be a means of maintaining face and managing one’s social impression or image, which is an activity that most individuals engage in Goffman (1959), Goffman (1955). The extrinsic motivation of avoiding being punished or judged (Ryan and Deci, 2020) drives study participants’ anticipated choice of engaging in HRI.

The relief of interacting with a non-judgmental robot with non-observant, meaning-laden eyes in a care setting has been previously acknowledged. The superpower of being non-judgmental is suggested to be a social power that allows for new, meaningful practices between machine-beings and humans and should serve as a starting point for designing HRI (Dörrenbächer et al., 2023a). Our study reports a sense of relief in the robot being non-judgmental and echoes the call for drawing on this power of the robot in design.

### 5.6 Alignment

We find that lexical and behavioral alignment, as well as *reciprocity*, is a powerful driver for study participants’ establishment of initial trust in the robot. The importance of *robot confirmation and acknowledgment* of human (lexical) input is a crucial requirement, as expressed by personas (Digging the) Dancing Queen and Teach Me How to Dance With You. Otherwise, feelings of insecurity and mistrust in the accuracy of the robot output arise, and coping mechanisms may kick in, such as changing one’s message to mitigate communication breakdowns. Adapting the way one talks to the robot impacts the experience of the dialog, which is in line with the claim of technological mediation theory that technology shapes how we behave (Verbeek, 2016). Earlier research has found that advanced linguistic dialog behaviors, such as lexical alignment (repeating what the user said), are, in fact, preferred and trusted more (Chiesurin et al., 2023). Our data hold many calls for echoing the input, functioning as a receipt of being understood. The persona in the tale of The (un-)Safety Dance showcases behavioral misalignment as she feels the robot does not behave as a human would, and therefore leads to feelings of uncertainty.

Another aspect of alignment is reciprocity in the dialog between the human and the robot, where both interlocutors give and take in communication characterized by adaptability and flexibility rather than in an indelicate, tactless dialog with interruptions and inconsideration. This finding is in line with earlier research, suggesting a positive relationship between reciprocity and trust in human–technology interactions (Gulati et al., 2019; Pinto et al., 2022).

## 6 Conclusion and future work

The socially assistive robot behavior in the study scenario disrupts how medication counseling processes could be carried out at pharmacies, dividing the task and responsibility between a pharmacist and a robot. Such a shift would bring about implications for customer behavior in and experience of the pharmacy. The findings illustrate the claim of technological mediation theory that technology organizes and shapes how we behave, our relationships, and how we experience the world. As the five themes in the form of song titles described above illustrate, the human–robot interaction shaped study participants’ experiences differently. Some study participants do not anticipate consulting a robot, should it be available for counseling about ECPs, due to mistrust and a preference for human-to-human interaction. Others might consult a robot, provided that several requirements are met. For them, the robot thus carries the potential to shape current practices of interacting with pharmacists, provided that the robot is trustworthy and safe to entrust with health data without harm or high cost to personal health. The six subthemes illustrate some of these requirements, reflecting the drivers of trust in this particular high-stakes scenario. In line with the idea that technology mediates how we experience things, the data show how Furhat is anticipated to shape the situation that informants role-played, for example, in terms of experiences of not being judged, more individual autonomy, more strain on how to speak and express oneself, increased feelings of uncertainty due to lexical and behavioral misalignment, and increased vulnerability as the robot’s physical position may signal to other customers what business the customer has.

The subthemes may, at first glance, seem disparate, but the data suggest that they are interlinked in a web of interdependencies. Factors serving as antecedents of trust do not only concern the robot or the human interacting with it but also the environment and the interaction between the robot and the human (Saßmannshausen et al., 2022). Our data seem to fall within all these four as the subthemes Shame and Autonomy relate to the human trustor, the subtheme Gaze to the robot trustee, Position to the pharmacy, and lastly, Alignment and Boundaries to the interaction between the interlocutors. We find interrelatedness, for example, in the link between how the gaze of the robot is perceived and experiences of shame in the medication counseling, as well as between informants’ calls for secure environments for the HRI (both physical and digital) and transparent, easy-to-understand communication of how the robot functions and behaves. Our analysis suggests that these determinants, in concert with many other factors, may then act as barriers or facilitators when establishing and calibrating trust in a robot in a similar use case, depending on their valence. A sustainable and ethical implementation of socially assistive robots in a pharmacy context must address these key aspects and requirements.

Our ambition is that the findings contribute to deepening the knowledge of trust factors at play in the interplay of HRI in the social context of a pharmacy and may inform future human-centered design work of socially assistive robot behavior and applications in pharmacy settings.

### 6.1 Study limitations

Several factors may be considered to limit the validity of the study findings. An inductive approach to data analysis risks being redundant and limited to surface descriptions and general summaries (Graneheim et al., 2017). Furthermore, the speculative approach may limit validity as we do not know whether the study participants have already purchased or may consider purchasing ECPs and, therefore, how they might relate to the scenario. The study participants merely speculate as to how they would behave; thus, they express attitudes and anticipations rather than reporting actions. Although intentions are considered a major determinant of behavior, the intention-behavior gap illustrates that future plans may not always be realized (Cheng and Cheng, 2023). Although the presence of a researcher at the rear of the room may have induced feelings of safety, should the robot malfunction, it may also limit or hamper the interaction and communication on a sensitive topic. The researcher was careful to stay in the background to balance this risk.

Additionally, the laboratory setting and a live interaction with an embodied, co-present robot might evoke different levels of trust than would studies in the wild or with a virtual or telepresent robot. Thus, our findings are not transferrable to other kinds of robots. Generalizability and transferability may be further hindered by the non-static, complex nature of the trust process. The experiences of a trustor are likely to change as a result of subjective factors, such as prior experience with robots, personality, individual and societal attitudes, and the context of the interaction, as well as robot behavior and levels of autonomy. Because trust is continuously recalibrated in time-/context-/people-sensitive circumstances, replications of the study may result in new and different findings. The transient state of the concept of trust in a socially assistive robot’s ability to meet human goals is one of many good reasons to advance the knowledge in the field. The aim of the study is not to obtain transferable insights into other populations but to explore anticipations, attitudes, and factors driving young adults’ decisions when establishing initial trust in a socially assistive robot providing medication counseling for ECPs. A limited sample size and the narrative approach through reflexive thematic analysis impede generalizability to all potential purchasers of ECPs in Finnish pharmacies. Nonetheless, portraying the study participants’ anticipations of and motives for trusting robot medication counseling offers insights into some of the trust dimensions at play.

Notwithstanding these limits, the merit of the study is found in a deeper understanding of the dimensions of the trust concept in the initial establishment or calibration phase, of driving trust factors, and of the subsequent anticipation to trust robots or not in a pharmacy context.

## Data Availability

The datasets presented in this article are not readily available because they contain non-anonymized data that may reveal participant identifiable data. Requests to access the datasets should be directed to SH, susanne.hagglund@abo.fi.
