# Supplementary material for: Do you wanna dance? Tales of trust and driving trust factors in robot medication counseling in the pharmacy context
Source: Front Robot AI. 2024 Aug 7;11:1332110. doi: 10.3389/frobt.2024.1332110 (PMC11336249; doi:10.3389/frobt.2024.1332110)
Supplement: Supplementary file 1 [file DataSheet1.pdf]

APPENDIX ONE

Scenario and task description

Customer – two days:

Imagine yourself visiting a pharmacy to buy a morning-after pill. You explain that you had unprotected intercourse two days ago. As a customer, you may require additional information about the medicinal product, and you may also have questions. Once you have acquired the product, you can leave the pharmacy.

Customer – four days:

Imagine yourself visiting a pharmacy to buy a morning-after pill. You explain that you had unprotected intercourse four days ago. As a customer, you may require additional information about the medicinal product, and you may have questions. Once you have acquired the product, you can leave the pharmacy.

APPENDIX TWO

Semi-structured interview guide

Age
Sex

Description of the personal experience of being met by a pharmacist compared to meeting a robot during a simulated pharmacy encounter

1. How was the experience different between **meeting** a pharmacist and meeting a robot in the simulated pharmacy encounter?
2. Did the **information** provided by the pharmacist differ from that given by the robot?
3. Did you ever feel **unsafe** during the meeting with the pharmacist or the robot?
4. Did you experience any differences in your **emotional state** when meeting the pharmacist compared to when meeting the robot?
5. Did you notice any differences in your ability to **be yourself** when meeting the pharmacist compared to when meeting the robot?

Description of future prospects / proposals

6. If you imagine a **reality** in which you are buying a morning-after pill at a pharmacy, would you prefer to be met by a pharmacist or a robot?
7. What would you **change** in the meeting with the pharmacist or the robot to ensure that the encounter would be a (an even) better experience?
